# Abnormal contractility in human heart myofibrils from patients with dilated cardiomyopathy due to mutations in TTN and contractile protein genes

**DOI:** 10.1038/s41598-017-13675-8

**Published:** 2017-11-01

**Authors:** Petr G. Vikhorev, Natalia Smoktunowicz, Alex B. Munster, O’Neal Copeland, Sawa Kostin, Cecile Montgiraud, Andrew E. Messer, Mohammad R. Toliat, Amy Li, Cristobal G. dos Remedios, Sean Lal, Cheavar A. Blair, Kenneth S. Campbell, Maya Guglin, Manfred Richter, Ralph Knöll, Steven B. Marston

**Affiliations:** 1 National Heart and Lung Institute, Imperial College London, London, W12 0NN United Kingdom; 2 Max-Planck-Institute for Heart and Lung Research, Ludwigstrasse 43, 61231, Bad Nauheim, 61231 Germany; 3 Cologne Center for Genomics, University of Cologne, Cologne, 50931 Germany; 4 Discipline of Anatomy and Histology, Bosch Institute, University of Sydney, Sydney, NSW 2006 Australia; 5 Division of Cardiovascular Medicine, Department of Physiology, University of Kentucky, Lexington, Kentucky USA; 6 Department of Cardiac Surgery, Kerckhoff-Clinic, Benekestrasse 2-8, Bad Nauheim, 61231 Germany; 7 ICMC (Integrated Cardio Metabolic Centre), Myocardial Genetics, Karolinska Institutet, University Hospital, Heart and Vascular Theme, Novum, Hiss A, våning 7, Hälsovägen 7-9, Huddinge, 141 57 Sweden; 8 AstraZeneca R&D Gothenburg, R&D, Innovative Medicines & Early Development, Cardiovascular, Renal and Metabolic Diseases (CVRM), Pepparedsleden 1, SE-431 83, Mölndal, Sweden

## Abstract

Dilated cardiomyopathy (DCM) is an important cause of heart failure. Single gene mutations in at least 50 genes have been proposed to account for 25–50% of DCM cases and up to 25% of inherited DCM has been attributed to truncating mutations in the sarcomeric structural protein titin (TTNtv). Whilst the primary molecular mechanism of some DCM-associated mutations in the contractile apparatus has been studied *in vitro* and in transgenic mice, the contractile defect in human heart muscle has not been studied. In this study we isolated cardiac myofibrils from 3 TTNtv mutants, and 3 with contractile protein mutations (*TNNI3* K36Q, *TNNC1* G159D and *MYH7* E1426K) and measured their contractility and passive stiffness in comparison with donor heart muscle as a control. We found that the three contractile protein mutations but not the TTNtv mutations had faster relaxation kinetics. Passive stiffness was reduced about 38% in all the DCM mutant samples. However, there was no change in maximum force or the titin N2BA/N2B isoform ratio and there was no titin haploinsufficiency. The decrease in myofibril passive stiffness was a common feature in all hearts with DCM-associated mutations and may be causative of DCM.

## Introduction

Historically, the prevalence of DCM was considered to be in the range of 1 in 2500–3000, however, newer modalities and heightened diagnostic awareness now estimate the prevalence of DCM of up to 1 in 500^1^. Dilated cardiomyopathy is inherited in about 25–50% of cases and more than 50 single genes are linked to inherited DCM, including genes encoding cytoskeletal, Z-disc, nucleo-cytoskeletal, mitochondrial, and calcium handling proteins making DCM a genetically heterogeneous disease. However “pure” inherited DCM, not associated with other symptoms, such as conduction disease, is most often caused by mutations in sarcomeric proteins. Recently titin truncating (TTNtv) mutations have been found in 25% of putative familial DCM (fDCM) cases^2^ and a disease causing role of these mutations in A band and Z-disk titin has been asserted^3^. Despite the emerging importance of TTNtv mutations as a cause of familial DCM (fDCM) very little is known about the functional effects of the mutations in human heart muscle.

In a previous study of human heart tissue samples we identified 26 potentially disease-causing mutations in 14 genes, 5 of which were in contractile proteins, 4 were in OBSCN and 6 were truncating mutations in the titin gene (TTNtv)^4^. The high frequency of potentially disease-causing mutations in end-stage DCM patients confirms the finding of Roberts *et al*., who studied a large population and demonstrated a >96% probability of pathogenicity in end-stage DCM patients with *TTN* truncation mutations^3^. In this study we have directly investigated TTNtv heterozygous mutations in human heart tissue samples in comparison with donor heart and DCM-causing mutations in other sarcomeric proteins. Several human samples were analysed on total titin expression level, N2BA/N2B isoform expression ratio, as well as titin phosphorylation level. To determine the mechanism of diastolic dysfunction, the maximum tension amplitude, length dependence of maximum force, and the rates of tension development and relaxation were measured in DCM samples carrying mutations in *TTN* (p. R23464T fs*41 and p. Y18923*), *TNNI3* (K36Q), *TNNC1* (G159D) and *MYH7* (E1426K).

## Results

### Samples and sequences

**Figure 1:**
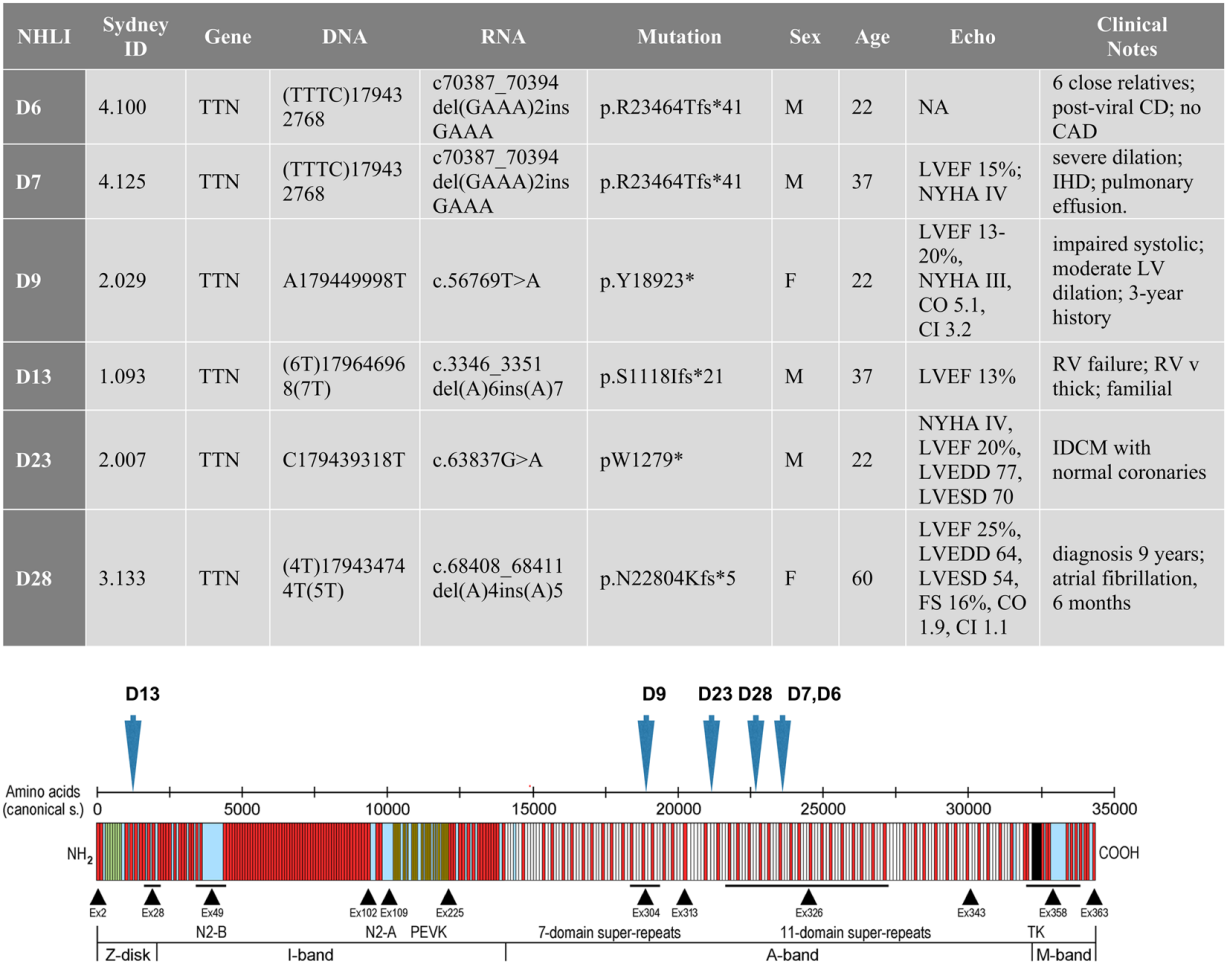
Identification of TTNtv mutations in human heart muscle. Clinical and genetic data on the heart muscle samples and location of mutations in the titin molecule, based on the model of Linke and Hamdani^29^. D6 and D7 have the same mutation but there is no evidence of their relationship except for common ethnicity. The calculated MW of TTNtv are 2611 kDa for D6/D7, 2102 kDa for D9, 125 kDa for D13, 2353 kDa for D23 and 2539 kDa for D28. Abbreviations: NA, not applicable; LVEF, left ventricular ejection fraction; NYHA, New York Heart Association; CO, cardiac output; CI, cardiac index; LVEDD, left ventricular end-diastolic diameter; LVESD, left ventricular end-systolic diameter; FS, fractional shortening; CD, cardiomyopathy; CAD, coronary artery disease; IHD, Ischaemic heart disease; LV, left ventricle; RV, right ventricle; IDCM, idiopathic cardiomyopathy.

A series of 30 explanted failing heart samples from selected patients with end-stage heart failure, most of which were diagnosed with familial DCM, was analysed by whole exome sequencing. We found six unique variants in the *TTN* gene predicted to cause chain termination that are potentially causative of DCM^4^. The presence of the variants was confirmed by direct sequencing (Fig. 1 and Supplementary Table S1).

### TTNtv mutations and contractile function

To investigate whether there was any mechanical dysfunction caused by putative DCM mutations we isolated cardiac myofibrils and measured their passive stiffness and contractility using an apparatus for single myofibril force studies. The three left ventricular TTNtv samples (D6, D7 and D9) were studied in comparison with myofibrils from donor heart and left ventricular muscle samples with mutations in contractile proteins of the sarcomere: troponin C (TnC) G159D, troponin I (TnI) K36Q and myosin heavy chain E1426K (D1, D2 and D15 respectively). These are termed familial DCM (fDCM) since linkage has been proven with these mutations^5–7^. Results are summarised in Table 1.

**Table 1:** Myofibril active force sarcomere length dependence, kinetics of force development and relaxation, and the parameters describing the passive mechanical myofibril properties. Data are shown as mean ± SEM.

	Sample ID	F_max_ at the SL shown below			Time-course parameters of myofibril activation and relaxation at SL 2.0 µm					Mechanical Parameters	
		1.8 µm (kPa)	2.0 µm (kPa)	2.2 µm (kPa)	k_ACT_ (s^−1^)	k_TR_ (s^−1^)	t_LIN_ (ms)	k_LIN_ (s^−1^)	k_REL_ (s^−1^)	Slack SL (µm)	*A*
Donor	KN1	47 ± 4 (10)	101 ± 7 (20)	109 ± 8 (13)	0.73 ± 0.06 (19)	0.63 ± 0.03 (19)	105 ± 7 (17)	0.44 ± 0.16 (17)	16.8 ± 1.7 (15)	1.89 ± 0.04 (9)	544 ± 66 (9)
	KN2	55 ± 8 (6)	102 ± 9 (7)	120 ± 16 (5)	0.91 ± 0.13 (7)	0.69 ± 0.07 (6)	86 ± 11 (6)	0.63 ± 0.18 (6)	18.08 ± 1.2 (6)	1.93 ± 0.03 (13)	529 ± 66 (13)
	NL	NA	NA	NA	NA	NA	NA	NA	NA	1.88 ± 0.04 (5)	561 ± 46 (5)
	NK	NA	NA	NA	NA	NA	NA	NA	NA	1.90 ± 0.04 (5)	663 ± 123 (5)
	Combined donors	50 ± 4 (16)	101 ± 5 (27)	112 ± 7 (18)	0.78 ± 0.06 (26)	0.64 ± 0.03 (25)	100 ± 6 (23)	0.49 ± 0.13 (23)	17.1 ± 1.2 (21)	1.91 ± 0.02 (32)	559 ± 38 (32)
fDCM	D1	58 ± 5 (4)	107 ± 10 (4)	134 ± 8 (4)	0.72 ± 0.06 (4)	0.62 ± 0.08 (4)	71 ± 6 (4)*	0.17 ± 0.07 (4)	20.0 ± 1.5 (3)	1.90 ± 0.03 (14)	329 ± 33 (14)***
	D2	40 ± 5 (9)	104 ± 11 (13)	123 ± 13 (11)	0.91 ± 0.10 (13)	0.67 ± 0.04 (13)	70 ± 5 (12)**	0.68 ± 0.28 (11)	22.32 ± 1.6 (12)*	1.94 ± 0.02 (21)	324 ± 36 (21)***
	D15	49 ± 6 (8)	88 ± 10 (10)	109 ± 7 (7)	0.88 ± 0.06 (11)	0.76 ± 0.06 (10)*	79 ± 7 (9)*	0.37 ± 0.20 (9)	23.1 ± 1.7 (9)*	1.90 ± 0.06 (6)	368 ± 41 (6)*
TTNtv	D6	52 ± 4 (6)	96 ± 6 (7)	114 ± 7 (7)	0.82 ± 0.05 (7)	0.68 ± 0.07 (7)	97 ± 7 (7)	0.52 ± 0.10 (7)	15.5 ± 0.8 (7)	1.86 ± 0.03 (18)	354 ± 37 (18)***
	D7	38 ± 4 (8)	98 ± 12 (8)	108 ± 7 (8)	0.87 ± 0.12 (8)	0.61 ± 0.05 (8)	98 ± 4 (8)	0.09 ± 0.19 (8)	19.3 ± 1.6 (8)	1.90 ± 0.02 (27)	367 ± 46 (27)***
	D9	52 ± 4 (17)	94 ± 4 (18)	116 ± 6 (18)	0.90 ± 0.07 (18)	0.74 ± 0.03 (18)*	98 ± 7 (17)	0.52 ± 0.6 (18)	20.0 ± 0.9 (17)	1.90 ± 0.03 (24)	348 ± 40 (24)***

The number of measurements obtained on different myofibrils is indicated in parentheses. *P < 0.05, **P < 0.01, ***P < 0.001 (one-way ANOVA analysis) vs combined donors. The fDCM samples have contractile protein mutations, D1 (*TNNC3* G159D), D2 (*TNNI1* K36Q) and D15 (*MYH7* E1426K). NA, data were not acquired.

**Figure 2:**
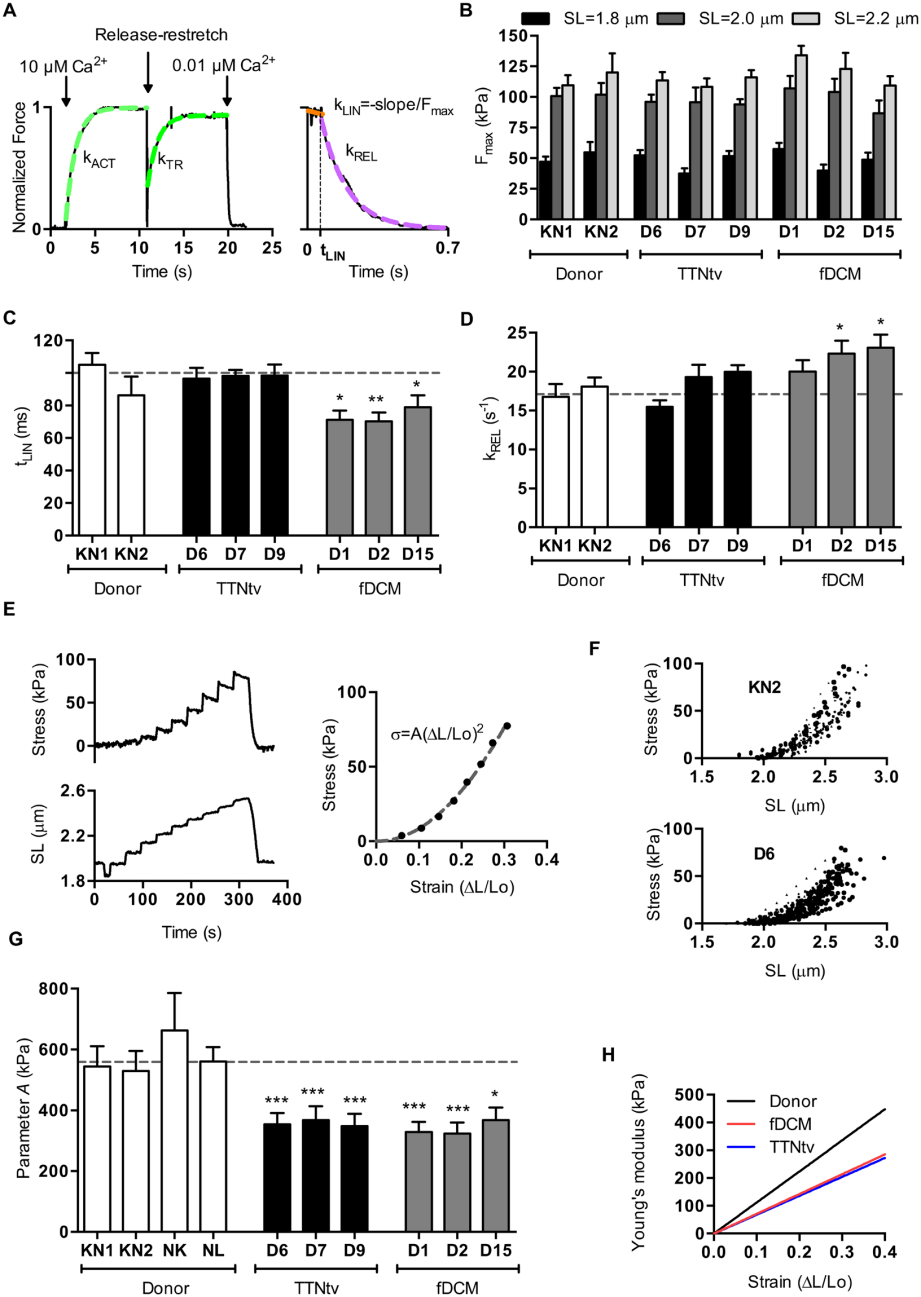
Myofibril contractility measurements. **(A)** The time course of contraction and relaxation in single myofibrils, showing the measured parameters. (**B**) Dependence of F_max_ on sarcomere length. Myofibril force was measured at sarcomere lengths of 1.8, 2.0 and 2.2 μm. (**C**) Effects of DCM-associated mutations on the kinetics of myofibril relaxation; duration of slow relaxation phase. (**D**) Effects of DCM-associated mutations on the kinetics of myofibril relaxation; rate of fast relaxation phase. **(E**) Myofibril passive elastic response. Tension trace (top) in response to incremental step changes in sarcomere length (bottom). The parabolic equation $\sigma (\varepsilon )=A{\varepsilon }^{2}$ was fitted (dashed line) to the stress-strain data. (**F**) Experimental data sets for samples KN2 and D6 plotted as stress versus sarcomere length. (**G**) Effects of DCM-associated mutations on the parameter *A*. (**H**) Effects of DCM-associated mutations on elastic modulus. The horizontal dotted lines in **C**, **D** and **G** indicate the means of the combined controls (2–4 donor hearts, n=21–32 myofibril samples). Data are shown as means ± SEM. *P < 0.05, **P < 0.01 and ***P < 0.001 (one-way ANOVA analysis) versus the combined controls.

Maximum isometric force depended on sarcomere length, being about maximal at 2.2 µm. The mean maximum force in the donor heart muscle myofibrils was 112 ± 7 kPa (n = 18), reduced to 50 ± 4 kPa (n = 16) at 1.8 µm sarcomere length (Fig. 2B and Supplementary Fig. S1). These values are comparable with previous measurements from ourselves and others^8,9^. In comparison, all the familial DCM muscle samples with contractile protein or TTNtv mutations produced the same maximum force at all three sarcomere lengths tested^10,11^, although there are some cases where force increases whilst Ca^2+^-sensitivity does not^12^.

Neither the activation (k_ACT_ and k_TR_) nor relaxation kinetics (t_LIN_, k_LIN_, k_REL_) were dependent on sarcomere length at maximally activating Ca^2+^ concentrations (Supplementary Fig. S1). This data suggests that sarcomere-length itself does not determine myosin crossbridge kinetics and number of force-generating myosin heads.

The rate of force increase following Ca^2+^ jump, k_ACT_, was 0.78 ± 0.06 s^−1^ (n = 26, SL = 2.0 µm) with the donor myofibrils, a value compatible with previous measurements, and was not significantly different in any of the DCM mutant heart myofibrils (TTNtv k_ACT_ = 0.86 ± 0.02 s^−1^, 3 heart samples; other fDCM k_ACT_ = 0.84 ± 0.06 s^−1^, 3 heart samples). k_TR_, which measures the rate of active crossbridge attachment, was 0.64 ± 0.03 s^−1^ (n = 25) for the donor heart myofibrils and very similar for the DCM mutant myofibrils (mean k_TR_ = 0.68 ± 0.04); there was a trend to a slightly higher k_TR_ in the mutant myofibrils that was statistically significant for D9 (1.16-fold higher) and D15 (1.18-fold higher). k_TR_ was consistently lower than k_ACT_, especially during the first contraction-relaxation cycle, however the difference was diminished with every new cycle. F_max_, after quick release-restretch, also did not fully recover. This shows that the state was progressively altered. Stretching may depress myofibril activation kinetics and force development in human cardiac myofibrils, but it is also possible that this effect could be related to a deterioration in the myofibril samples (Table 1).

Myofibrillar relaxation has a lag phase of almost constant force followed by an exponential decrease in force (Fig. 2A). In donor heart samples the duration of the slow phase was 100 ± 6 ms and the rate of the fast relaxation phase was 17.1 ± 1.2 s^−1^. The TTNtv mutation myofibrils had rates of relaxation indistinguishable from donor but the three contractile protein fDCM mutations relaxed about 27% faster (Fig. 2C and D).

We then measured passive stiffness. Myofibrils in relaxing solution containing 30 mM BDM were stretched in several cumulative steps. We found that the slack sarcomere length was 1.91 ± 0.02 µm (n = 32) in the donor heart samples and was not significantly different in any of the mutant samples. The increase in passive tension with length was less than donor for all of the mutant samples. For the donor heart samples, the parameter *A* was 559 ± 38 kPa (n = 32), corresponding to a Young’s modulus of 170 kPa at 2.2 µm sarcomere length (Fig. 2G and H). We found substantial reductions in passive stiffness in all of the fDCM samples including the TTNtv mutants. On average the fDCM myofibrils had a 38% lower passive stiffness (Young’s modulus) (Fig. 2H).

### Troponin function in TTNtv mutant hearts

Previous studies of heart samples with fDCM-related mutations in thin filament proteins have indicated that the troponin is abnormal, with a consistent loss of modulation of myofilament Ca^2+^-sensitivity by PKA-mediated phosphorylation of TnI at Ser 22 and 23 and it has been proposed that this uncoupling is sufficient to induce the DCM phenotype^13,14^. We therefore investigated the Ca^2+^-regulatory properties of phosphorylated and unphosphorylated troponin isolated from ventricular or atrial muscle using the *in vitro* motility assay. Donor heart troponin showed the normal modulation of Ca^2+^-sensitivity. Troponin from the heart samples with mutations in contractile proteins was uncoupled; this has been previously shown for *TNNI3* K36Q and *TNNC1* G159D *in vitro*^14–16^, but uncoupling due to a myosin heavy chain mutation (*MYH7* E1426K) is novel. Three TTNtv heart samples showed normal modulation of Ca^2+^-sensitivity by TnI phosphorylation, while D6 and D7 showed a blunted relationship of TnI phosphorylation to Ca^2+^-sensitivity (Supplementary Fig. S2 and Table S2). This suggests that it is unlikely that un-coupling could be a primary cause of the DCM phenotype associated with titin mutations.

### Titin expression and modification in human heart

**Figure 3:**
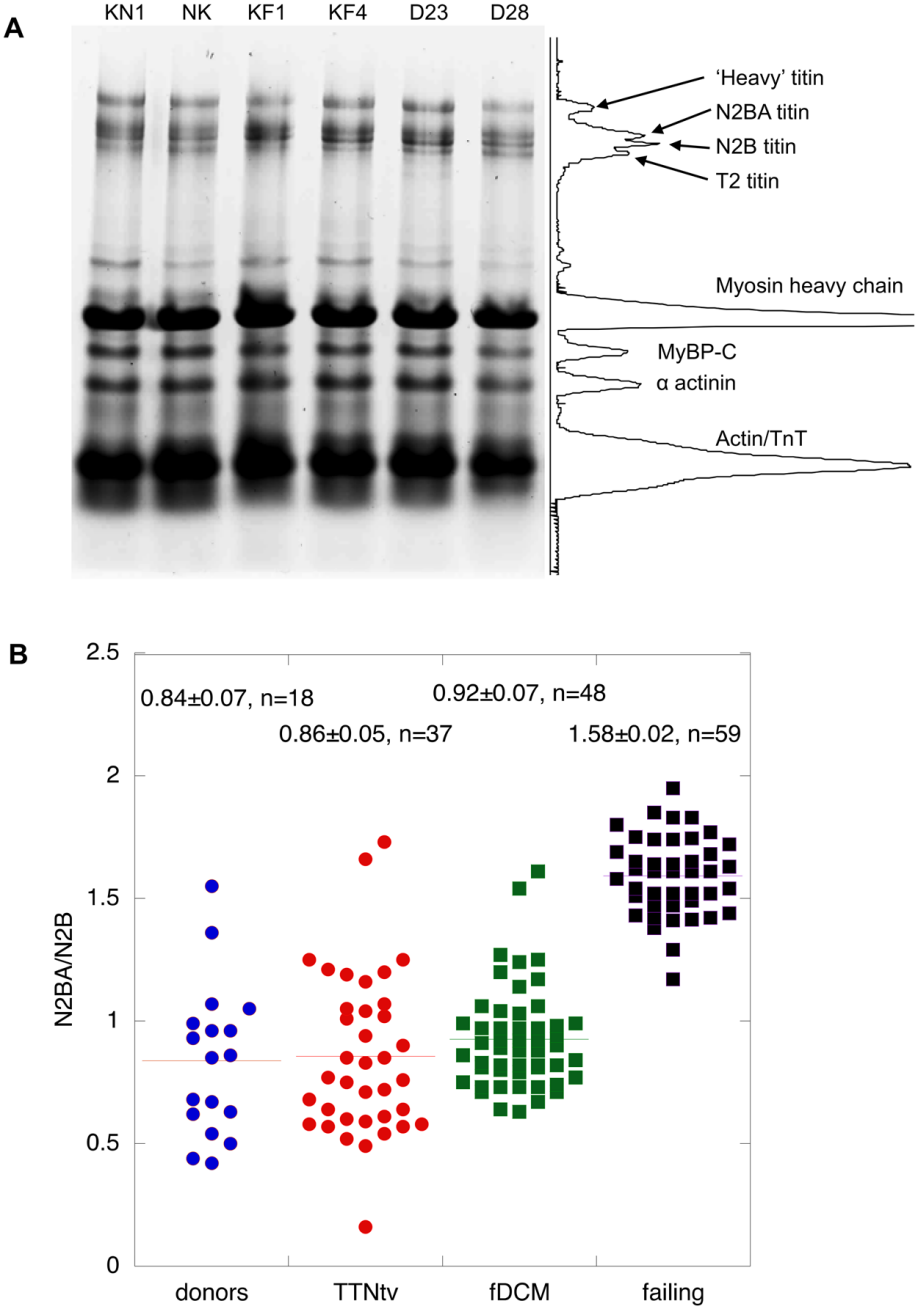
Titin isoform expression. (**A**) 2% agarose/2% polyacrylamide-SDS gel electrophoresis of human heart myofibrils showing separation of titin isoforms, identified in the scanned profile: Two donors, KN1, NK, two failing heart, KF1, KF4, and two TTNtv mutant samples are shown. (**B**) Comparison of N2BA/N2B ratio measured in donors, TTNtv, other fDCM and idiopathic DCM. Pooled data from multiple gels similar to **A**. Data shown for donor heart represents KN1 (17 replicates), NK (13 replicates) and NH (8 replicates). Data shown for TTNtv represents D6 (7 replicates), D7 (8 replicates), D9 (6 replicates), D23 (15 replicates), D28 (15 replicates) and D29 (4 replicates). Data for other fDCM represents D1 (5 replicates), D2 (8 replicates), D4 (4 replicates), D11 (9 replicates), D12 (9 replicates) and D15 (9 replicates). Data shown for idiopathic failing heart represents FG (13 replicates), FH (11 replicates), FI (7 replicates), KF1 (8 replicates), KF2 (3 replicates), KF3 (9 replicates) and KF4 (12 replicates). Only idiopathic DCM samples have a significantly higher N2BA/N2B ratio than donor (1.9x).

**Figure 4:**
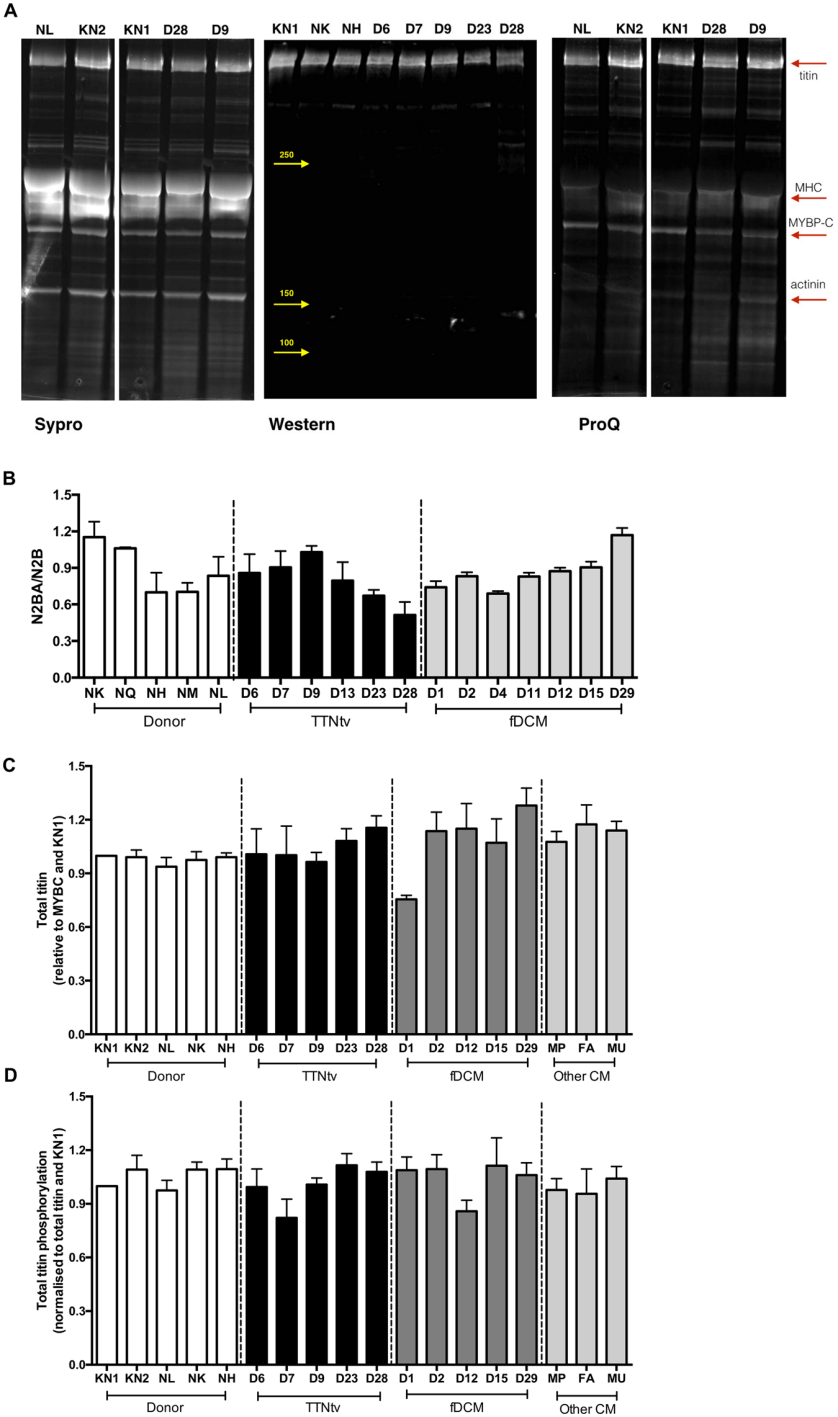
Analysis of titin expression and phosphorylation. (**A**) Representative 4–20% SDS-PAGE gels of myofibrillar fraction or whole tissue extracts of human heart muscle showing titin. Left: The myofibrillar fraction stained with SYPRO Ruby protein stain. Middle: Western blots of whole tissue extracts were probed with 9D10 anti-titin antibody. Right: The myofibrillar fraction counterstained with Pro-Q Diamond phosphoprotein stain. Plots **B**, **C** and **D** are based on densitometry of a large number of replicate gels. Yellow arrows indicate positions of MW markers, red arrows indicate myofibrillar proteins. (**B**) N2BA/N2B titin isoform expression ratio was estimated from 4–20% SDS-PAGE. (**C**) Total titin protein level normalised to MyBP-C was assessed by SYPRO Ruby staining of 4–20% SDS-PAGE. The titin bands were identified by Western blotting. (**D**) The total titin phosphorylation level was assessed from Pro-Q Diamond staining relative to Sypro Ruby stain. Gel to gel variations were normalised to KN1 sample, loaded in all gels. The bars show the means and SEM for replicate measurements on the same sample (n = 3–10). No significant differences were between the means of the donor group and the means of the patient groups (one-way ANOVA, P > 0.05).

In idiopathic heart failure it has been noted that the proportion of the less stiff N2BA isoform relative to N2B is increased and may therefore account for the reduced passive stiffness observed^17,18^. We found that there were no significant differences between the N2BA/N2B ratio of donor heart or any of the mutant DCM heart muscle samples, although we did observe a higher ratio in myofibrils from idiopathic DCM as expected (Figs 3 and 4).

The study of Roberts *et al*. likewise found the same N2BA/N2B ratio in donor and TTNtv mutant muscle^3^. We also investigated the possibility of reduced titin expression. Using methodology similar to that which previously identified obscurin haploinsufficiency, we compared donor heart myofibrils with the TTNtv samples and also with the other mutant DCM samples and found no significant haploinsufficiency either in myofibrils or whole tissue extracts^19^ (Fig. 4). Finally, using Pro-Q Diamond staining for phosphorylation that successfully identified hypophosphorylation of titin in HFpEF^20^ we found no systematic differences in the phosphorylation level of titin from donor, TTNtv or other mutant DCM myofibrils (Fig. 4). This finding does not exclude the possibility of equal and opposite changes of phosphorylation at the individual phosphorylation sites of titin.

### Morphological findings

**Figure 5:**
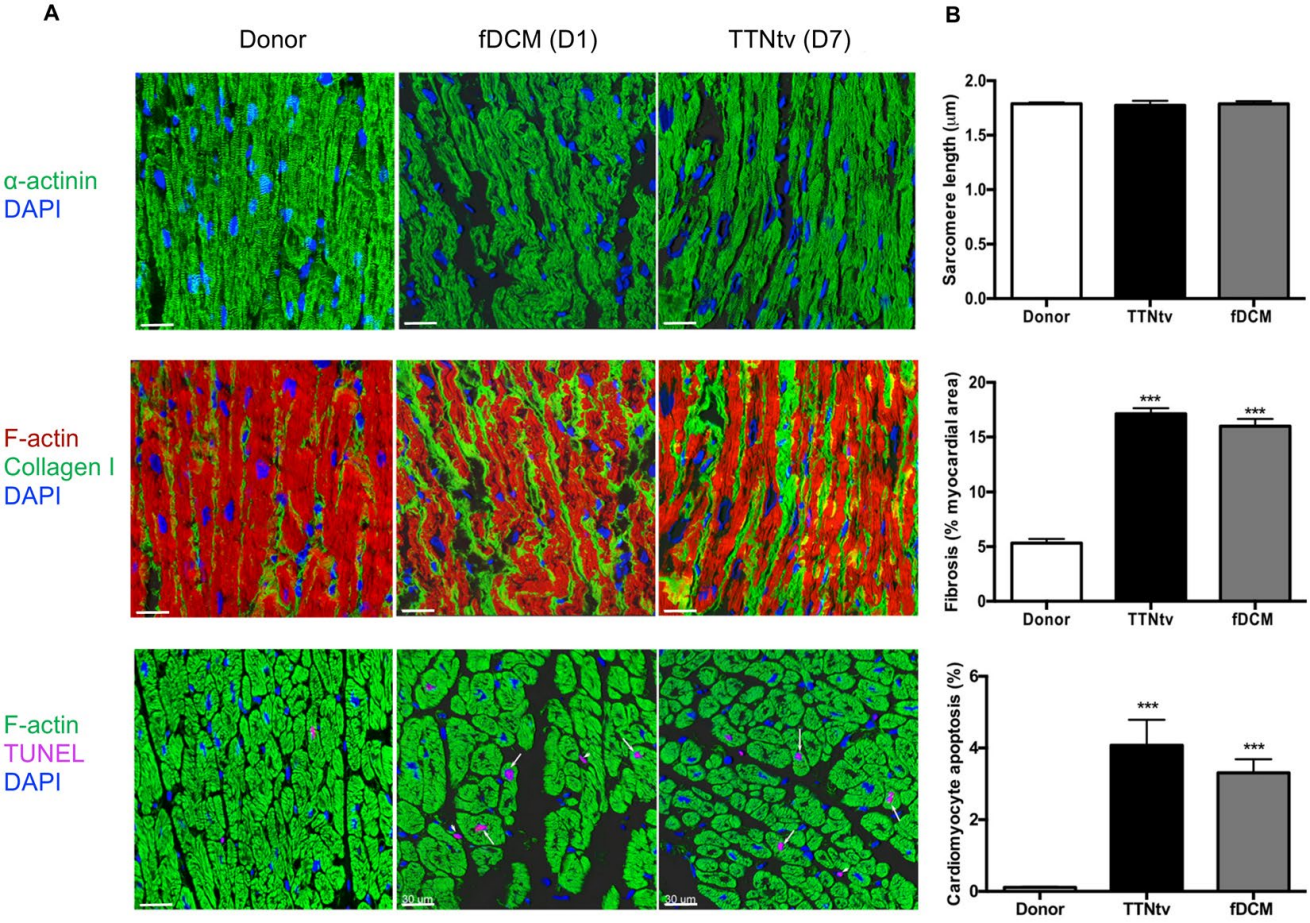
Immunofluorescence microscopy of TTNtv and fDCM heart muscle samples. **(A)** Examples of immunofluorescent microscopy using antibodies against α-actinin to show sarcomere structure, collagen to show fibrosis, and TUNEL to show apoptosis. The scale bars are 30 µm. (**B**) Quantification of sarcomere length, fibrosis and apoptosis (see Supplementary Table S3). Data are shown as means ± SEM, One-way ANOVA analysis, ***P < 0.001.

In ultrastructure studies, interstitial fibrosis was significantly increased in all the DCM samples compared to donor hearts, and TUNEL analysis showed strongly increased apoptosis of cardiomyocytes in all the fDCM samples (Fig. 5 and Supplementary Table S3). Comparison of the sarcomere length in donor hearts with that in DCM heart samples did not show any consistent differences.

## Discussion

We have performed a functional analysis of sarcomeric protein mutations using explanted tissue from patients with a diagnosis of fDCM. The mutations in TnC, TnI and myosin heavy chain have been studied *in vitro* and the familial dilated cardiomyopathy phenotype is well established^5–7,15^. In this study we have measured contractility and passive stiffness in human cardiac myofibrils with the sarcomeric fDCM mutations and also putative fDCM due to TTN truncating mutations^3^.

Our measurements of the rate of donor myofibril activation are similar to those previously published; we observed a fast relaxation rate similar to^9^ but considerably faster than reported by Poggesi’s group^8^; the differences probably relate to different experimental conditions such as phosphate concentration, Ca^2+^ concentration and temperature.

The direct measurements of contractile function show that the DCM-related mutations studied here can be distinguished from human heart tissue with hypertrophic cardiomyopathy (HCM). In contrast to the studies on human heart tissue from HCM patients^21^, we found the maximum isometric force was not diminished, and length dependent activation of maximal force was not altered in any of the DCM mutants. This is consistent with our detailed measurements using myofibrils from the *ACTC* E361G DCM transgenic mouse^11,21–23^.

It is also apparent that the contractile protein fDCM and TTNtv mutant phenotypes are distinct from idiopathic DCM or HFpEF since neither N2BA/N2B ratios or contractile protein and titin phosphorylation levels are different from donor heart^3,17–19,24^.

It is important to note that the changes in contractile properties of adult human tissue samples with TTNtv mutations are very different from TTNtv mutations incorporated into iPSC-derived cardiomyocytes which exhibit sharply reduced contractility, haploinsufficiency, expression of truncated peptides and decreased sarcomere length^25^, none of which were observed in our adult myofibril samples. The different abnormalities in TTNtv hiPSC-CM and adult myofibrils is most likely due to the immaturity of iPSC-derived cardiomyocytes which functionally resemble foetal muscles^26^. In contrast, the properties of muscle from transgenic mice with a heterozygous TTNtv mutation^19,27^ and our human heart samples are comparable, suggesting that human iPSC-derived cardiomyocytes may indicate congenital effects of TTNtv mutations but do not reproduce the disease-causing process of DCM due to TTNtv observed in adult patients.

It is noteworthy that the DCM-associated mutations in contractile proteins are functionally distinct from DCM-related TTNtv mutant tissue. The faster relaxation rates of the contractile protein mutations D1, D2 and D15 suggest a reduced Ca^2+^-sensitivity, since k_REL_ increases with increasing EC_50_^11^. This was not observed for the TTNtv mutations. In addition, uncoupling provides another potential causative mechanism for contractile protein mutations^14,28^. Several recent studies have demonstrated that mutations in thin filament proteins lead to uncoupling of TnI phosphorylation by protein kinase A from the change in Ca^2+^-sensitivity measured in isolated thin filaments and myofibrils. This leads to a blunting of the adrenergic response that was demonstrated to be sufficient to cause DCM under stress^13,14,28^. Whilst the contractile protein mutations in samples D1, D2 and D15 showed uncoupling, uncoupling was not a consistent feature of the TTNtv mutations suggesting that it is unlikely that uncoupling contributes to the DCM phenotype associated with titin truncating mutations.

These studies confirm the expectation that familial DCM caused by mutations in different genes can work through a number of different mechanisms that cause loss of function, in contrast to the apparent single mechanism for the gain of function in HCM.

The most striking abnormality found in our samples was a 38% reduced passive stiffness for all the fDCM samples. In the case of the TTNtv mutations this was the only functional abnormality we detected and is thus likely to be indicative of the disease-causing abnormality. Our data showing a decrease in passive stiffness are in accord with other studies, notably of samples from idiopathic DCM hearts, and it has been proposed that this phenomenon could be explained by an increased expression of the N2BA *TTN* isoform or altered titin phosphorylation levels^17,18,20^. In our fDCM and TTNtv samples we observed reduced passive stiffness with no difference in isoform expression (N2BA/N2B or presence of foetal isoforms) and no haploinsufficiency; nor was there incorporation of predicted truncated peptides into the myofibrils in common with other researchers^3,19^. There was no significant difference in overall titin phosphorylation level in the fDCM and TTNtv samples but we cannot exclude a change in the relative phosphorylation at sites in titin that enhance stiffness (PKC) and sites that reduce stiffness (PKA, PKG)^29^. At present we have no explanation for why the fDCM mutations cause reduced passive stiffness other than the possibility of reduced Ca^2+^-sensitivity may promote relaxation. For the TTNtv mutations it is possible that the mutations cause changes in exon usage that modulate elasticity as was observed with *RBM20* ablation^30^ whilst causing no detectable change in molecular size. It is likely that the decreased passive stiffness would have an effect when the heart muscle is shortening under load that would not be apparent in the isometric contraction^31^. Such changes could be related to disruption of the dynamics of myosin head activation by stretch leading to reduced contractility^32^ or alteration of the “slow force response” by modulating of mechanosensitive signalling pathways that affect [Ca^2+^]i homeostasis and promote apoptosis and fibrosis as observed in our samples^33^. In concert such processes could impair contractility and potentially trigger a DCM phenotype.

## Materials and Methods

### Human heart samples

We used anonymised tissue samples from explanted hearts in the Sydney Heart Bank^34^. Twenty-eight patients with a diagnosis of familial or idiopathic DCM requiring a heart transplant at a young age were selected for sequencing and further studies of which 13 are listed in Supplementary Table S1. As controls we studied muscle from eight donor hearts and two myectomy samples from patients with HCM. Donor hearts were obtained when no suitable transplant recipient was found. The donors had no history of cardiac disease, the hearts showed no structural cardiac pathology and had normal electrocardiogram and the heart ventricular function. Donor heart samples KN1 and KN2 were from University of Kentucky^35^. Patients were consented under ethical approvals obtained from the Australian Red Cross Blood Service (donor hearts) (HREC #2012/2814) and St Vincent’s Hospital, Darlinghurst, the University of Sydney (failing hearts: HREC #H91/048/1a; donor hearts: HREC #2016/923), and the University of Kentucky, USA (08–03338-F2L). The collection and use of control samples used for immunofluorescence microscopy was approved by the ethics committee of the Medical Council of the State of Hessen. The investigations conform to the principles of the Declaration of Helsinki. The functional characteristics of some of the donor heart and myectomy samples have been previously reported^24,36^ (see Supplementary Table S1).

### Sequencing and gel electrophoresis

DNA extraction, whole exome sequencing, was performed as previously reported^4,24,37–39^.

### Pyrosequencing

TTNtv mutations were confirmed by standard pyrosequencing according to manufacturer’s instructions on a PSQ™ 96 HS instrument (Qiagen, Hilden, Germany) and analyzed by pyrosequencing software.

### Gel electrophoresis

Whole tissue extracts were obtained for gel electrophoresis using T-Per Protein extraction reagent (Thermo Scientific 78510) including 1 µg/ml E-64, chymostatin and leupeptin protease inhibitors according to the manufacturer’s protocol. The myofibrillar fraction of heart muscle was prepared by our standard protocol^24^. For the best resolution myofibril samples were dissolved in gel buffer without heating. High molecular weight proteins were separated on 4–15% SDS-PAGE gradient gels (Criterion, Bio-Rad) with a running time of 2.5 hours at 100 volts or on 2% agarose/2% polyacrylamide “titin gels”.

To assess protein phosphorylation 4–15% SDS-PAGE gradient gels were stained with Pro-Q Diamond phosphoprotein specific stain followed by SYPRO Ruby total protein stain. Western blots were made in a wet blotting apparatus for 5 hours at 250 milliamps at 4 °C. Blotted proteins were reversibly stained with MemCode Reversible stain kit (Thermo Scientific) and then probed with 9D10 monoclonal antibody to titin, specific to the I band region^38^ (Developmental Studies Hybridoma Bank, Iowa) and visualised with ECL plus (GE Healthcare).

### Titin gel preparation and electrophoresis

The method was slightly adapted from that of Tatsumi & Hattori^39^. Agarose (0.3 g) was stirred in 20 mls of deionized water and dissolved by heating in a microwave for 1 min. The molten agarose was poured into a pre-warmed falcon tube (water bath set at 50 °C) and left to cool. The acrylamide solution was prepared as follows: 17.1 ml (deionized water), 3.6 ml 22.2% acrylamide (acrylamide: N,N’-methylenebiscarylamide = 20:1), 4 ml gel buffer (0.4 M tris, 0.2 M sodium acetate, 20 mM EDTA, 1% SDS, pH 7.4) and 15% ammonium persulphate, 200 µl (mixed in this order). The mixture was warmed in the water bath for 5 mins. 13.3 ml of the cooled agarose was added to the acrylamide solution and gently mixed, 30 µl of TEMED was added to the acrylamide/agarose mix and gently inverted. The gel solution was rapidly poured into a glass gel cassette (approx. 80 × 75 × 1.5 mm) and the combs were added instantly. The gels were then cooled at 4 °C for 7–10 min, then returned to room temperature for 2 hours. The samples were loaded and the gel was run at 4 °C at 7.5 mA for 30 mins, then raised to 15 mA (total time about 4 hours).

### Manipulation and measurement of TnI phosphorylation level

Troponin isolated from human heart samples was treated to change the level of phosphorylation. They were dephosphorylated by treatment with shrimp alkaline phosphatase (Sigma, P9088) or phosphorylated by treatment with protein kinase A (PKA) catalytic subunit (Sigma, P2645–400) as previously described^14,40^. TnI phosphorylation level in isolated troponin was measured by phosphate affinity SDS-PAGE as described by Messer *et al*.^41^.

### Quantitative *in vitro* motility assay

Troponin isolated from human heart samples was treated to change the level of phosphorylation (see above). Thin filaments were reconstituted with 10 nM rabbit skeletal muscle α-actin (labelled with TRITC phalloidin), tropomyosin (40–60 nM) and troponin (60 nM) to study Ca^2+^-regulation of filament motility^14,42^. Thin filament movement over a bed of immobilised rabbit fast skeletal muscle heavy meromyosin (100 μg/ml) was compared in dual channel motility cells in which troponin varied by a single factor (mutation or phosphorylation state). The temperature was set to 29 °C. Filament movement was recorded and analysed as previously described^43^, yielding two parameters, the fraction of filaments moving and the speed of moving filaments. The fraction motile and the sliding speeds were measured over a range of Ca^2+^ concentrations to generate Ca^2+^-activation curves as shown previously^14,44^. The data were fitted to the 4-variable Hill equation to yield a value for EC_50_ and n_H_.

### Mechanical measurements on myofibrils

The apparatus and methodology for the measurement of force and passive stiffness in single myofibrils has been described^45^. Single myofibrils were isolated from samples of human left ventricular tissue^45^ and kept on ice for use in experiments for up to 3 days. Myofibrils were isolated from the human heart samples and suspended horizontally using specially prepared microneedles. Contraction and relaxation were initiated by a rapid Ca^2+^ concentration jump achieved by a fast-solution switch system implementing a double-barrelled micropipette moving across the mounted myofibril. Relaxing (0.01 µM Ca^2+^ or no added Ca^2+^) and activating (10 µM Ca^2+^) solutions contained (mM): MOPS 10, MgATP 5, free Mg^2+^ 1, DTT 5, phosphocreatine 10, creatine kinase (200 Units/mL), bacterial purine nucleoside phosphorylase (0.1 Units/mL), 7-methylguanosine 0.3, pH 7.0. The Ca-EGTA: EGTA ratio was set to obtain 10 mM total EGTA and the desired free [Ca^2+^]. Potassium propionate and sodium sulphate were added to adjust the ionic strength of the solution to 200 mM. The relaxing solution in the bath chamber was supplemented with (in μM): chymostatin 10, pepstatin 5, leupeptin 40, E-64 10, PMSF 200. The rate of force increase at 10 µM Ca^2+^, the maximum achieved isometric force, the rate of force regain after a quick release and re-stretch (k_TR_) and the slow and fast phase component rates of relaxation (t_LIN_, k_LIN_ and k_REL_) at 0.01 µM Ca^2+^ were measured. Additionally, 30 mM of 2,3-butanedione monoxime (BDM) was added to the relaxing solution with no added Ca^2+^ during myofibril passive stiffness measurements. The experiments were performed at 17 °C.

Data were fitted using Levenberg-Marquardt nonlinear least square algorithm in LabVIEW. The rate constants for the exponential force development (k_ACT_) and redevelopment (k_TR_) and for the fast phase of relaxation (k_REL_) were evaluated by curve-fitting using the equation $\,y(t)\,=\,{y}_{plateau}+({y}_{0}-{y}_{plateau})exp(-kt)$. The slow phase was fit with a linear fit and characterized by the rate constant k_LIN_ calculated from the slope value of the linear fit by normalizing it to the maximum tension (${k}_{LIN}=-{\rm{slope}}/{F}_{max}$). A parabola $\sigma (\varepsilon )=A{\varepsilon }^{2}$ was fit to the stress-strain data, where $\sigma$ is stress and ε is strain ($\varepsilon (L)=(L-{L}_{0})/{L}_{0}$, where ${L}_{0}$ is slack sarcomere length). Young’s modulus was determined as the derivative of the function and is defined by the equation $\,E(\varepsilon )=2A\varepsilon$. Statistics analysis (one-way ANOVA) and graphs were prepared using GraphPad Prism 6 (GraphPad Software, San Diego, CA).

### Immunofluorescence microscopy

For these studies control tissues consisted of: LV samples from four patients subjected to subvalvular myectomy during surgical aortic valve replacement, and LV samples from two donor hearts with normal LV function which could not be used for transplantation. Patients with aortic valve stenosis showed preserved cardiac function (ejection fraction ≥ 60%), and the resected myocardial tissue did not present signs of ischemic damage or myocardial hypertrophy.

5 μm cryosections were air dried and fixed with 4% paraformaldehyde. A rabbit polyclonal primary antibody against collagen I (Rockland) and a mouse monoclonal antibody against sarcomeric α-actinin (clone EA-53, Sigma) were used. Secondary detection system consisted of anti-rabbit or anti-mouse IgG and streptavidin linked Cy 3 (Amersham). TRITC Phalloidin was purchased from Sigma and used to stain F-actin; DAPI (nuclear staining) was purchased from Vector Laboratories. Apoptosis was analyzed using the *in situ* Cell Death Detection Kit (Roche, #116847959110) according to the manufacturer’s instructions. Tissue sections were examined by laser scanning confocal microscopy (Leica TCS SP2 and Leica TCS SP8). A series of confocal optical sections were taken using a Leica Plan Apo × 63/1.32 objective lens. After data acquisition, the images were transferred to a Windows 7 workstation for restoration and three-dimensional reconstruction using Imaris 7.7 multichannel image processing software (Bitplane, Zürich, Switzerland) described below.

### Analysis of confocal microscopy

Quantification of collagen I was performed as described previously^46,47^. In brief, cryosections from at least two different levels in each tissue sample were used. All samples were immunolabeled simultaneously with identical conditions of fixation and dilutions of primary and secondary antibodies. Sections exposed to PBS instead of primary antibodies served as negative controls. For each heart at least 10 random fields of vision were analyzed using image analysis (Leica) and Image J software (NIH). The quantification process was performed blinded to the type of section, having on the screen only one channel showing F-actin labelling. For each quantification, a specific confocal setting was established and kept constant in all measurements. Quantification was performed by measurements of fluorescence intensity by using a range of 0 to 255 grey values. The area of collagen I was calculated as percent of positive labelling per tissue area. Quantification of apoptotic cells was performed as described^48^. Sarcomere length was measured by intensity profiles of α-actinin in XYZ axes using the three-dimensional extended “Section” mode of the Imaris 7.7 program.

## Electronic supplementary material

Supplementary Information
