# Author Correction: Abnormal contractility in human heart myofibrils from patients with dilated cardiomyopathy due to mutations in TTN and contractile protein genes

**DOI:** 10.1038/s41598-018-32408-z

**Published:** 2018-09-24

**Authors:** Petr G. Vikhorev, Natalia Smoktunowicz, Alex B. Munster, O’ Neal Copeland, Sawa Kostin, Cecile Montgiraud, Andrew E. Messer, Mohammad R. Toliat, Amy Li, Cristobal G. dos Remedios, Sean Lal, Cheavar A. Blair, Kenneth S. Campbell, Maya Guglin, Manfred Richter, Ralph Knöll, Steven B. Marston

**Affiliations:** 1 National Heart and Lung Institute, Imperial College London, London, W12 0NN United Kingdom; 2 Max-Planck-Institute for Heart and Lung Research, Ludwigstrasse 43, 61231, Bad Nauheim, 61231 Germany; 3 Cologne Center for Genomics, University of Cologne, Cologne, 50931 Germany; 4 Discipline of Anatomy and Histology, Bosch Institute, University of Sydney, Sydney, NSW 2006 Australia; 5 Division of Cardiovascular Medicine, Department of Physiology, University of Kentucky, Lexington, Kentucky USA; 6 Department of Cardiac Surgery, Kerckhoff-Clinic, Benekestrasse 2-8, Bad Nauheim, 61231 Germany; 7 ICMC (Integrated Cardio Metabolic Centre), Myocardial Genetics, Karolinska Institutet, University Hospital, Heart and Vascular Theme, Novum, Hiss A, våning 7, Hälsovägen 7-9, 141 57, Huddinge, Sweden; 8 AstraZeneca R&D Gothenburg, R&D, Innovative Medicines & Early Development, Cardiovascular, Renal and Metabolic Diseases (CVRM), Pepparedsleden 1, SE-431 83, Mölndal, Sweden

Correction to: *Scientific Reports* 10.1038/s41598-017-13675-8, published online 01 November 2017

Manfred Richter was omitted from the author list in the original version of this Article.

The Acknowledgements section now reads:

“This work was supported by grants from the British Heart Foundation (RG/11/20/29266 and PG/17/5/32705). R.K. was supported by the Fondation LeDucq. We thank the patients and staff of St. Vincent’s Hospital Sydney, Kerckhoff Heart Center, Bad Neuhein, Germany, and the University of Kentucky Hospital, Lexington, KY.”

The Author Contributions section now reads:

“P.G.V. designed and performed the single myofibril studies and did the data analysis. M.R.T. and R.K. did the sequencing. C.M., A.B.M., N.S. and O.C. performed the Western blots and gel electrophoresis. N.S. and A.E.M. performed the in vitro motility assay experiments. S.K. did the confocal microscopy. C.G.d.R., A.L., M.R., C.B., M.G. and K.S.C. provided the material. S.B.M. and R.K. designed the study. All authors wrote and reviewed the manuscript.”

In addition, the original version of this Article contained a typographical error in the spelling of Ralph Knöll, which was incorrectly given as Ralph Knoll.

Furthermore, Affiliation 7 was incorrectly given as ‘Integrated Cardio Metabolic Centre, Karolinska Institutet, Myocardial Genetics, Karolinska University Hospital in Huddinge, Huddinge, 14186, Sweden’.

Finally, the original version of this Article omitted an affiliation for Ralph Knöll. The correct affiliations for Ralph Knöll are listed below:

National Heart and Lung Institute, Imperial College London, London, W12 0NN, United Kingdom

ICMC (Integrated Cardio Metabolic Centre), Myocardial Genetics, Karolinska Institutet, University Hospital, Heart and Vascular Theme, Novum, Hiss A, våning 7, Hälsovägen 7-9, 141 57 Huddinge, Sweden

AstraZeneca R&D Gothenburg, R&D, Innovative Medicines & Early Development, Cardiovascular, Renal and Metabolic Diseases (CVRM), Pepparedsleden 1, SE-431 83 Mölndal, Sweden

These errors have now been corrected in the PDF and HTML versions of the Article, and in the accompanying Supplementary Information file.

