# Supplementary material for: Abnormal contractility in human heart myofibrils from patients with dilated cardiomyopathy due to mutations in TTN and contractile protein genes
Source: Sci Rep. 2017 Nov 1;7:14829. doi: 10.1038/s41598-017-13675-8 (PMC5665940; doi:10.1038/s41598-017-13675-8)
Supplement: Supplementary file 1 — Supplementary Information [file 41598_2017_13675_MOESM1_ESM.pdf]

SUPPLEMENTARY INFORMATION

Abnormal contractility in human heart myofibrils from patients with dilated cardiomyopathy due to mutations in TTN and contractile protein genes.

Petr G. Vikhorev*, Natalia Smoktunowicz, Alex B. Munster, O'Neal Copeland, Sawa Kostin, Cecile Montgiraud, Andrew E. Messer, Mohammad R. Toliat, Amy Li, Cristobal G. dos Remedios, Sean Lal, Cheavar A. Blair, Kenneth S. Campbell, Maya Guglin, Manfred Richter, Ralph Knöll, Steven B. Marston*

*To whom correspondence should be addressed: Email: p.vikhorev@imperial.ac.uk (P.G.V.); s.marston@imperial.ac.uk (S.B.M.)

SUPPLEMENTS:

Supplemental Figures:
Fig. S1. Sarcomere length dependence of contractile parameters.
Fig. S2. Troponin function measured by in vitro motility assay.
Supplemental Tables:
Table S1. Donor and patient data for the samples used in this study.
Table S2. Troponin function measured by in vitro motility assay.
Table S3. Quantitative analysis of confocal microscopy.
Supplemental References.

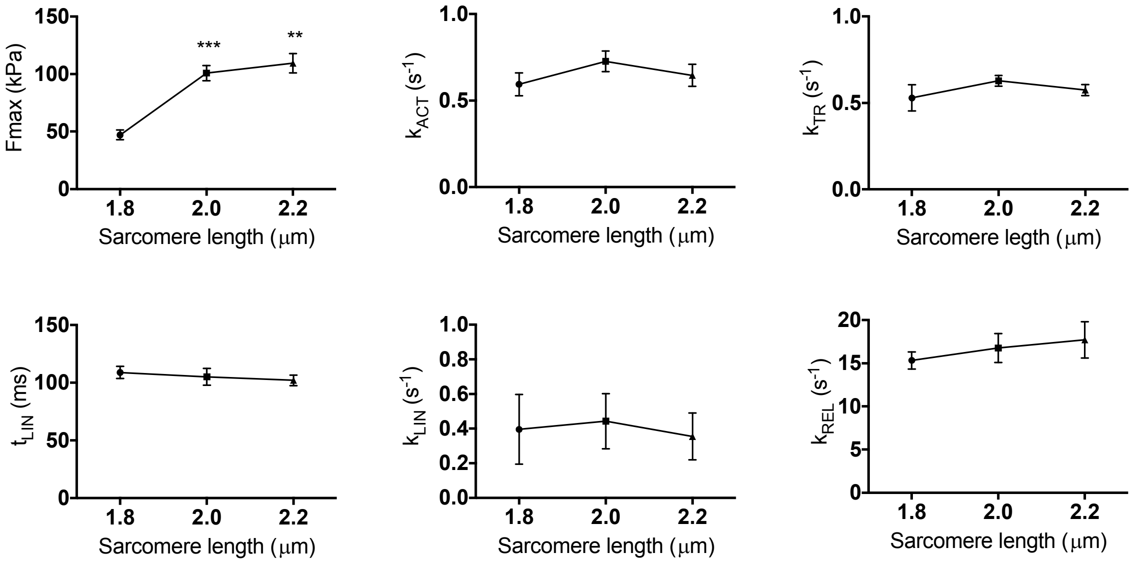

Figure S1. Dependence of myofibril force development on sarcomere length. The maximum force depends on sarcomere length but the time course of force development (k_{ACT}, k_{TR}) and relaxation (t_{LIN}, k_{LIN}, k_{REL}) do not show length dependence. The data are for the control donor heart sample KN1. Myofibrils were activated and relaxed by step changes in Ca^{2+} concentration achieved by moving a double-barrelled micropipette across the mounted myofibril.

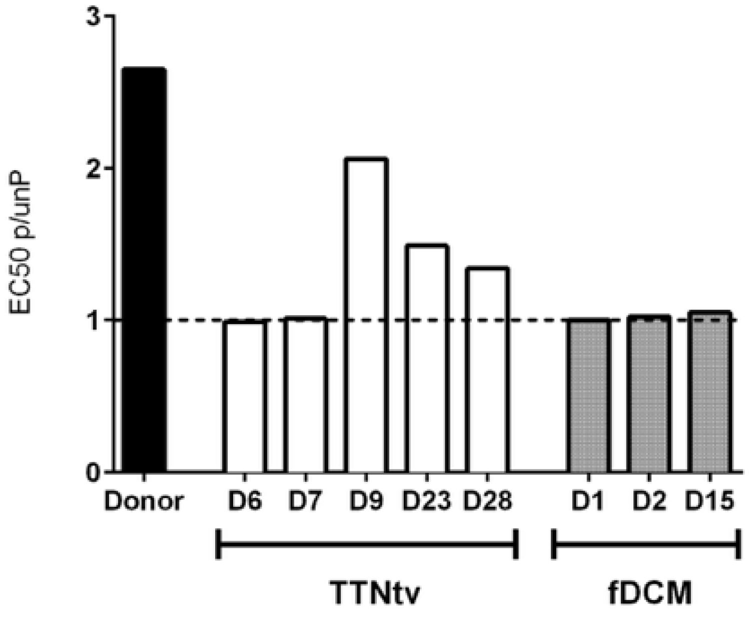

Figure S2. Troponin function measured by *in vitro* motility assay. EC_{50} of phosphorylated relative to unphosphorylated is plotted. Normally EC_{50} of phosphorylated thin filaments is 2-3 fold higher than unphosphorylated. When uncoupled the ratio is 1. Full data in table S2.

Supplemental Tables:

Table S1. Donor and patient data for the samples used in this study. ID shows the code for the samples in the Sydney Heart bank, * indicates code for samples from University of Kentucky. The data for explanted putative familial DCM hearts in this table is reproduced from Marston et al (2015)[2]. Abbreviations: SAH subarachnoid haemorrhage, HBI hemorrhagic brain injury, ICH intracerebral haemorrhage, fDCM familial dilated cardiomyopathy, IDCM idiopathic dilated cardiomyopathy, IHD ischemic heart disease, HCM hypertrophic cardiomyopathy, LV left ventricle, RV right ventricle, IVS interventricular septum.

	ID	Gene	Mutation	Diagnosis	Sex	Age	Heart chamber	EF	Ref.
**Donor**									
**KN1**	24713*			Head trauma	F	47	LV		
**KN2**	CF462*			ICH	F	61	LV		
**NL**	5.131			SAH	F	42	LV		
**NK**	5.128			SAH	M	45	LV		
**NH**	5.089			SAH	F	48	LV		3
**NM**	5.138			HBI	M	23	LV		3
**NS**	7.08			SAH	F	55	LV		
**NQ**	5.003			ICH	M	37	RV		3
**Failing Heart (Genetic)**									
**D1**	-	TNNC1	pG159D	fDCM	M	3	LV	20%	4
**D2**	2.008	TNNI3	pK36Q	fDCM	M	15	LV, RV	10-20%	5
**D4**	2.066	OBSCN<br>DSP	pE963K<br>pR1537C	fDCM	M	43	LV	10%	2,6
**D6**	4.100	TTN	pR23464T fs*41	IDCM	M	22	LV	15%	
**D7**	4.125	TTN	pR23464T fs*41	fDCM	M	37	LV	15%	
**D9**	2.029	TTN	pY18923*	fDCM	F	22	LV	13-20%	
**D11**	4.121		None found	fDCM	F	29	LV	20%	
**D12**	4.047	MYOM1	pE247K	fDCM	F	63	LV	20%	
**D13**	1.093	TTN	p1118I fs*21	IDCM	M	37	RV	13%	
**D15**	3.111	MYH7	pE1426K	fDCM	M	43	LV	17%	7
**D23**	2.007	TTN	pW21279*	IDCM	M	22	RA	20%	
**D28**	3.133	TTN	pN22804K fs*5	fDCM	F	60	RA	25%	
**D29**	4.066	PKP2	pI531S	fDCM	M	52	RA	7-15%	8
**HCM**									
**MP**	-			HCM	F	62			
**MU**	-	MYH7	L427M	HCM	M	63			
**Failing heart (idiopathic)**									
**FA**	4.032			IDCM	M	31	LV	10-15%	
**FG**	5.057			IDCM	M	52	LV	10-17%	
**FH**	5.066			IDCM	F	34	LV	30%	
**FI**	3.167			IDCM	M	47	LV	20%	
**KF1**	74B28*			IDCM	F	66	LV	25%	
**KF3**	117DD*			IDCM	F	56	LV	21.5%	
**KF4**	73CE5*			IDCM	F	65	LV	15%	

Table S2. Troponin function measured by *in vitro* motility assay. Ca^{2+} sensitivity of thin filaments containing troponin from donor and mutant heart muscle at high (>1.5 mol Pi/mol TnI) and low (<0.3) phosphorylation levels was measured by *in vitro* motility assay. Mean and SEM (n=1-6) is shown. EC_{50} of mutant relative to donor and of phosphorylated relative to unphosphorylated is also plotted in Fig. S2.

	Donor	D1	D2	D15	D6	D7	D9	D23	D28
**EC_{50} P (µM)**	0.067±0.009 (n=4)	0.08±0.01 (n=5)	0.18±0.03 (n=5)	0.03±0.002 (n=3)	0.04±0.02 (n=5)	0.03±0.01 (n=6)	0.059±0.03 (n=5)	0.065 (n=1)	0.046 (n=1)
**EC_{50} unP (µM)**	0.026±0.004 (n=4)	0.08±0.02 (n=5)	0.18±0.04 (n=5)	0.03±0.01 (n=3)	0.04±0.02 (n=5)	0.03±0.01 (n=6)	0.03±0.01 (n=5)	0.043 (n=1)	0.034 (n=1)
**EC_{50} mutant /donor**	NA	1.19	2.7	0.44	0.58	0.4	0.87	0.97	0.68
**EC_{50} P/unP**	2.65	0.97±0.03	1.02±0.05	1.05±0.04	0.99±0.17	1.01±0.04	2.06±0.35	1.49	1.34
**Ref.**		From Dyer *et al.* 2009 [9]	From Papadaki *et al.* 2015 [10]						

Table S3. Quantitative analysis of confocal microscopy. The fraction of cardiomyocytes and interstitial cells undergoing apoptosis was determined from TUNEL microscopy. The % of area occupied by interstitial fibrosis was determined from the collagen I antibody signal and sarcomere length was measured directly. See supplementary materials and methods. Summary results are plotted in Fig. 5.

Sample	Diagnosis	Age	Sex	Cardiomyocyte apoptosis (%)	Interstitial cell apoptosis (per 1 mm^2)	Fibrosis (% myocardial area)	Sarcomere length (µm)
**C1**	Donor	45	F	0.047	0.092	3.92	1.82
**C2**	Donor	43	M	0.056	0.139	4.72	1.79
**C3**	AS	55	M	0.115	0.394	6.56	1.74
**C4**	AS	49	N	0.192	0.421	6.07	1.77
**C5**	AS	59	M	0.135	0.179	5.17	1.77
**C6**	AS	52	M	0.127	0.194	5.44	1.81
**D1**	DCM	3	M	2.89	4.79	14.66	1.79
**D2**	DCM	15	M	3.41	3.62	16.56	1.73
**D15**	DCM	45	M	3.62	4.22	16.74	1.82
**D6**	DCM/TTNtv	22	M	4.39	4.11	18.10	1.71
**D7**	DCM/TTNtv	37	M	2.72	3.59	16.88	1.75
**D9**	DCM/TTNtv	22	F	5.12	3.88	16.3	1.85
**t-test, controls vs DCM**				P<0.001	P<0.001	P<0.001	P=0.7479

Supplemental References:

1 Linke, W. A. & Hamdani, N. Gigantic Business: Titin Properties and Function Through Thick and Thin. *Circ Res* **114**, 1052-1068, (2014).

2 Marston, S. *et al.* OBSCN Mutations Associated with Dilated Cardiomyopathy and Haploinsufficiency. *PLoS One* **10**, e0138568, (2015).

3 Mollova, M. *et al.* Cardiomyocyte proliferation contributes to heart growth in young humans. *Proc Natl Acad Sci U S A* **110**, 1446-1451, (2013).

4 Kaski, J. P., Burch, M. & Elliott, P. M. Mutations in the cardiac Troponin C gene are a cause of idiopathic dilated cardiomyopathy in childhood. *Cardiol Young* **17**, 675-677 (2007).

5 Carballo, S. *et al.* Identification and functional characterization of cardiac troponin I as a novel disease gene in autosomal dominant dilated cardiomyopathy. *Circ Res* **105**, 375-382 (2009).

6 Xu, T. *et al.* Compound and digenic heterozygosity contributes to arrhythmogenic right ventricular cardiomyopathy. *J Am Coll Cardiol* **55**, 587-597, (2010).

7 Villard, E. *et al.* Mutation screening in dilated cardiomyopathy: prominent role of the beta myosin heavy chain gene. *Eur Heart J* **26**, 794-803, (2005).

8 den Haan, A. D. *et al.* Comprehensive Desmosome Mutation Analysis in North Americans With Arrhythmogenic Right Ventricular Dysplasia/Cardiomyopathy. *Circ: Cardiovasc Genet* **2**, 428-435, (2009).

9 Dyer, E. *et al.* Functional Analysis of a Unique Troponin C Mutation, Gly159Asp that Causes Familial Dilated Cardiomyopathy, Studied in Explanted Heart Muscle. *Circ Heart Fail* **2**, 456-464, (2009).

10 Papadaki, M., Vikhorev, P. G., Marston, S. B. & Messer, A. E. Uncoupling of myofilament Ca2+ sensitivity from troponin I phosphorylation by mutations can be reversed by epigallocatechin-3-gallate. *Cardiovasc Res* **108**, 99-110, (2015).
